# Targeting epigenetic regulators to boost T cell immunotherapy against cancer

**DOI:** 10.1186/s13578-026-01533-y

**Published:** 2026-02-03

**Authors:** Tiaojiang Xiao, Ying E. Zhang

**Affiliations:** Laboratory of Cellular and Molecular Biology, Center for Cancer Research, National Cancer Institute, NIH, Building 37, RM 2056, Bethesda, MD 20892-4256 USA

**Keywords:** T-cell based immunotherapy, Epigenetic regulator, Chromatin modification, Tumor microenvironment (TME)

## Abstract

Epigenetic regulation is crucial in directing T-cell differentiation, function, and fate, thereby influencing the success of T-cell-based immunotherapies. This review begins with an overview of the evolution of T-cell immunotherapies in cancer treatment. We then examine key epigenetic regulators—such as DNA methylation, mRNA methylation, and histone methylation—and their roles in shaping T-cell states during infection and tumorigenesis. The contributions of these regulators to T-cell exhaustion and lineage commitment are discussed in the context of immunotherapy efficacy. We highlight recent advances in targeting epigenetic pathways to enhance CAR-T and TCR-based therapies and conclude with current challenges and emerging strategies to improve the durability and effectiveness of adoptive T-cell therapies.

## Introduction

T cell-based immunotherapies have revolutionized cancer treatment by harnessing the body's immune system to selectively target and eliminate malignant cells. These therapies rely on the ability of T cells to recognize tumor antigens and mount a cytotoxic response; a concept rooted in decades of immunological research. Advances in genetic engineering have made it possible to modify T cells to enhance their specificity, persistence, and functionality, leading to the clinical success of adoptive T cell therapies [77, 95].

Among the most prominent T cell-based immunotherapies are chimeric antigen receptor (CAR)-T cell therapy and T cell receptor (TCR)-based therapy. CAR-T cells are engineered to express synthetic receptors that recognize tumor-associated antigens independent of major histocompatibility complex (MHC) presentation. This approach has demonstrated remarkable efficacy in treating hematological malignancies, particularly B-cell leukemias and lymphomas. In contrast, TCR-based therapies leverage the natural T cell receptor to recognize intracellular tumor antigens presented by MHC molecules, offering a promising strategy for solid tumors. While these therapies have achieved significant clinical success, their effectiveness in solid tumors remains limited due to multiple challenges [88, 100].

A major obstacle in T cell-based immunotherapy is the immunosuppressive tumor microenvironment (TME), which actively inhibits T cell function through metabolic constraints, inhibitory ligands, and immunosuppressive cell populations such as regulatory T cells (Tregs) and myeloid-derived suppressor cells (MDSCs). Additionally, T cell exhaustion, driven by chronic antigen exposure, results in the progressive loss of T cell effector functions and persistence. Tumor cells further evade immune recognition through mechanisms such as MHC downregulation and the secretion of immunosuppressive exosomes, making it difficult for engineered T cells to sustain an effective antitumor response [47, 67].

To overcome these challenges, manipulating epigenetic modifications has emerged as a promising strategy to enhance T cell fitness, persistence, and resistance to immune suppression. Epigenetic modifications, including DNA methylation, histone modifications, and RNA methylation, regulate gene expression without altering the genetic code and play a crucial role in shaping T cell differentiation and function. Recent studies suggest that targeting these epigenetic pathways can reverse exhaustion and enhance the efficacy of adoptive T cell therapies, with DNA methyltransferase inhibitors (DNMTis) and other epigenetic drugs showing preclinical success [123].

While various studies have explored histone acetylation, ubiquitination, phosphorylation, and non-coding RNAs in T-cell therapies [4, 5, 70, 84], this review focuses specifically on methylation, including DNA, mRNA, and histone methylation, as a central regulatory mechanism. We first examine the role of these methylations in T-cell function and exhaustion during infection and tumorogenesis, then highlight recent advances in targeting methylation-related pathways to enhance CAR-T and TCR-based therapies. Finally, we discuss current challenges and future directions for incorporating epigenetic strategies into next-generation adoptive T-cell therapies to improve clinical outcomes.

## The evolution of T cell-based immunotherapies in cancer treatment

The concept of harnessing the immune system to fight cancer began with the discovery of T cells as central players in immune defense. In the 1960s, Drs. Max Cooper and Jacques Miller identified T and B cells as key components of adaptive immunity, establishing the role of T cells in recognizing and eliminating abnormal cells, including tumor-infiltrating lymphocytes [20, 79]. However, tumors were later found to evade immune surveillance and suppress T-cell activity, limiting their natural antitumor potential. This challenge prompted researchers to enhance T-cell function for therapeutic purposes.

In the late 1980s and early 1990s, scientists developed the first generation of chimeric antigen receptor (CAR) T cells by engineering synthetic receptors that enabled T cells to recognize and kill tumor cells independently of MHC [81, 132]. Although this innovation was groundbreaking, it did not produce durable anti-tumor responses. Around the same time, T cell receptor (TCR)-based therapies also emerged, aiming to target intracellular tumor antigens presented on MHC molecules. Early TCR approaches showed potential but were limited by poor persistence and modest clinical efficacy [75].

During the early 2000s, advances in CAR-T cell therapy led to the development of second- and third-generation CARs that included costimulatory domains such as CD28 and 4-1BB (also known as CD137). These enhancements improved T cell activation, memory formation, and persistence [110]. Meanwhile, early TCR therapies demonstrated clinical responses in melanoma and sarcoma by targeting melanocyte differentiation antigens like MART-1 and cancer-testis antigens such as NY-ESO-1. Despite these advances, TCR therapy remained constrained by the need for patient-specific HLA matching, prompting research into universal TCRs and affinity-enhanced receptors [19].

In 2011, immune checkpoint blockade (ICB) entered clinical use with the FDA approval of ipilimumab (anti-CTLA-4), followed by pembrolizumab and nivolumab (anti-PD-1) in 2014 [38, 57]. These agents reactivated exhausted T cells and significantly improved survival in cancers like melanoma. However, the limited efficacy of ICB and CAR-T in solid tumors, due to the immunosuppressive tumor microenvironment (TME), antigen loss, and poor T cell trafficking, fueled interest in combining these therapies with other approaches.

A major milestone occurred in 2017 with the FDA approval of tisagenlecleucel (Kymriah) for relapsed or refractory B-cell acute lymphoblastic leukemia (ALL), marking the first CAR-T therapy approved for clinical use. The therapy achieved a 76% overall survival rate at 12 months [59]. Subsequent approvals for B-cell lymphomas and multiple myeloma validated CAR-T therapy’s success in hematologic malignancies.

By the 2020s, new strategies expanded the reach of T cell-based immunotherapies. Researchers developed therapies targeting glypican proteoglycans overexpressed in liver cancers, using both antibody-based and T cell-based approaches [64, 133]. At the same time, gene editing tools such as CRISPR-Cas9 enabled precise modifications to T cells, enhancing their specificity, persistence, and resistance to exhaustion. Epigenetic reprogramming also emerged as a promising strategy to boost T cell function and memory.

Today, CAR-T and TCR therapies continue to evolve. Combination strategies involving checkpoint inhibitors, metabolic modulators, and epigenetic agents are being explored to improve efficacy, especially in solid tumors. By integrating genetic engineering and immunomodulation, researchers aim to overcome current limitations and advance the next generation of T cell-based cancer therapies.

## Roles of DNA methylation in infection and tumorigenesis

DNA methylation is an epigenetic modification involving the enzymatic addition of a methyl group to the 5th carbon of cytosine residues. This modification most frequently targets cytosines within CpG dinucleotides. It is commonly found in CpG islands (CGIs), which are dense clusters of CpGs located near transcriptional start sites (TSSs). Contrary to repression, the CGIs associated with actively transcribed genes are typically unmethylated, a state crucial for active gene expression. Repression of an associated gene occurs when a CGI becomes hypermethylated, most notably observed in the silencing of tumor suppressor genes in cancer. DNA methylation is also found in lower densities across other genomic regions, including CGI shores (regions 0–2 kb from CGIs), CGI shelves (regions 2–4 kb from CGIs), and Intergenic non-coding regions. Methylation within gene bodies has been linked to transcriptional activation [10, 48]. Therefore, to fully understand DNA methylation patterns, it is crucial to extend analyses beyond promoters and TSSs to explore diverse genomic contexts.

In mammals, DNMT3A, DNMT3B and DNMT3L are responsible for establishing de novo DNA methylation, while DNMT1 ensures the maintenance of methylation patterns during DNA replication [105, 111]. DNA methylation typically results in gene silencing by promoting a condensed chromatin structure that hinders transcription factor binding and RNA polymerase activity [80]. This modification is also critical for maintaining genomic stability, regulating gene expression, and ensuring proper cellular differentiation and function. Furthermore, DNA methylation is a dynamic process, with reversible modifications achieved through active or passive mechanisms, including the activity of ten-eleven translocation (TET) enzymes that convert 5-methylcytosine (5mC) to oxidized derivatives like 5-hydroxymethylcytosine (5hmC) [135].

T cell development and differentiation rely heavily on tightly regulated DNA methylation to shape lineage commitment and functional specialization. Thymocytes in the thymus undergo significant gene expression changes, guided by DNA methylation, to form mature T cell subsets including naïve, CD4⁺ helper, and CD8⁺ cytotoxic T cells [94]. After exiting the thymus, T cells further diversify into effector and regulatory subsets in response to antigenic stimulation and cytokine cues. Naïve CD4⁺ T cells differentiate into Th1, Th2, Th17, and T follicular helper (Tfh) lineages, each driving distinct immune programs ranging from cellular immunity to humoral and pro-inflammatory responses. Regulatory T cells (Tregs), defined by FOXP3 expression, maintain tolerance and limit inflammation, while CD8⁺ T cells become cytotoxic lymphocytes capable of eliminating infected or malignant cells [27, 60]. The lineage identity and functional stability of each subset are reinforced by specific DNA methylation patterns, ensuring appropriate immune responses and preventing dysregulated activation [49].

### DNA methylation during acute infection: supporting effector differentiation and memory formation

#### Clonal expansion and effector gene induction

Acute infection triggers a rapid and highly coordinated process in which naïve CD8⁺ T cells undergo massive clonal expansion and differentiate into short-lived effector cells (SLECs) capable of clearing pathogens. This transition is driven not only by antigenic stimulation and inflammatory cues but also by extensive remodeling of the DNA methylation landscape [93]. Naïve T cells are characterized by a largely methylated chromatin state at key effector loci including *PRF1* (Perforin), *GZMB* (Granzyme B), and *IFNG* (Interferon-γ), which maintains these genes in a transcriptionally silent configuration [130].

Upon activation, DNMT activity is locally reduced at these effector-promoting regions, and TET-mediated demethylation becomes dominant. Enhancers and promoters of effector genes progressively lose methylation, permitting transcription factor binding and initiating high-level expression of cytotoxic effector molecules. This demethylation is both rapid and stable, serving as a hallmark of effector differentiation and enabling the acquisition of robust pathogen-clearing functionality. Disruption of this demethylation process, even transiently, impairs cytotoxic gene induction and compromises early pathogen control, demonstrating the functional significance of DNA methylation remodeling in effector T-cell responses [21, 36].

#### Transition to memory: the poised epigenetic state

Following pathogen clearance, most effector T cells undergo apoptosis; however, a small subset differentiates into long-lived memory T cells. Memory cells occupy a unique epigenetic state that is neither fully naïve nor fully effector but rather “poised.” Their methylomes preserve selective demethylation at key effector loci such as *IFNG*, *PRF1*, and *GZMB*, even though these genes are minimally expressed at rest. This poised demethylated configuration enables rapid transcriptional reactivation upon secondary antigen encounter, producing the hallmark rapid and robust recall responses of memory T cells [2, 129].

Memory precursors are also characterized by preservation of the transcription factor TCF-1 (encoded by *TCF7*), whose promoter and regulatory elements must remain hypomethylated to sustain expression. TCF-1 promotes stem-like features, supports self-renewal, and enables the long-term survival of memory populations. Consequently, the methylation state of *TCF7* serves as a determinant of memory lineage fidelity [23, 134].

#### Role of DNMTs in maintaining effector-to-memory balance

The maintenance DNA methyltransferase DNMT1 is essential for stabilizing methylation patterns during T-cell division. Genetic deletion of *DNMT1* in activated T cells results in aberrant hypomethylation, diminished effector differentiation, impaired clonal expansion, and defective memory formation. The inability to retain effector methylation signatures disrupts normal T-cell fate transitions, illustrating that appropriately calibrated methylation rather than simple hypo- or hypermethylation is necessary for effective immune memory. Thus, DNA methylation serves as both a permissive and restrictive mechanism, balancing effector gene induction with the preservation of memory-forming potential [21].

### Chronic infection and cancer: epigenetic imposition of T-cell exhaustion

During chronic viral, bacterial, or parasitic infection, T cells experience persistent antigenic stimulation and sustained inflammatory signals, including NF-κB, STAT3, IL-6, and IL-1 pathways. These cues progressively reshape the T-cell epigenome by inducing DNMT1, DNMT3A, and DNMT3B activity. Effector and memory programs become distorted as key loci required for cytokine production, cytotoxicity, and proliferation acquire aberrant DNA methylation, while inhibitory-receptor genes often remain unmethylated and transcriptionally active. In parallel, chronic inflammation can drive hypomethylation in repetitive or oncogenic regions, promoting genomic instability in surrounding tissues. These durable methylation “scars” lock T cells into dysfunctional, exhaustion-prone states and contribute to a tumor-permissive microenvironment, thereby linking long-term infection to impaired immunity and increased carcinogenesis [1, 90, 91, 128].

#### DNMT3A and the establishment of exhaustion

Among the de novo methyltransferases, DNMT3A plays a crucial role in establishing and reinforcing exhaustion-specific methylation patterns. Chronic antigen exposure leads to sustained DNMT3A expression, which progressively methylates effector-associated loci, such as *PRF1*, *GZMB*, *IFNG*, *lL2*, and others, thereby silencing their transcription. These methylation marks create a durable repressive program that is resistant to reversal even when antigen levels decline [50, 85, 87].

Notably, conditional deletion of *DNMT3A* in activated T cells prevents the acquisition of terminal exhaustion. T cells deficient in DNMT3A retain greater effector potential, express higher levels of cytotoxic molecules, and exhibit increased proliferative capacity, even in the presence of chronic antigen [3]. These findings demonstrate that DNMT3A-dependent methylation is a primary mechanism driving the irreversible transition to exhaustion.

#### Sustained expression of inhibitory receptors through demethylation

While effector genes become hypermethylated and silenced during exhaustion, genes encoding inhibitory receptors show the opposite trend. The *PDCD1* locus (encoding PD-1) becomes demethylated and remains stably open, driving persistent high-level expression of PD-1. This demethylated state persists even after antigen withdrawal or PD-1 blockade therapy, contributing to the long-term stability of the exhausted phenotype[90].

Thus, exhaustion is characterized by two complementary methylation regimes: (1) Hypermethylation of effector loci → transcriptional repression; (2) Hypomethylation of inhibitory loci → constitutive inhibitory receptor expression. This dichotomy establishes the stable transcriptional architecture that defines exhausted T-cell identity.

#### Implications for immune checkpoint blockade (ICB)

The epigenetic rigidity of terminally exhausted T cells has major implications for immunotherapy. ICB therapies targeting PD-1 or CTLA-4 aim to reinvigorate dysfunctional T cells; however, only a specific subset of progenitor exhausted T cells responds robustly. These cells retain expression of TCF-1, preserve a less locked methylation landscape, and maintain the proliferative capacity necessary to expand upon checkpoint blockade. In contrast, terminally exhausted cells harbor extensive DNMT3A-mediated repressive methylation marks that cannot be reversed by ICB, making them largely unresponsive [138].

The distinction between these two exhausted subsets highlights the central role of DNA methylation in determining therapeutic outcomes. It also underscores the need for strategies that modify methylation to restore T-cell responsiveness in chronic infection and cancer [33].

### DNA methylation in the tumor microenvironment (TME): balancing effector and immunosuppressive forces

Within the TME, the outcome of T-cell responses is shaped not only by exhaustion but also by the balance between effector T cells, regulatory T cells (Tregs), and CD4⁺ effector subsets such as Th1 cells. DNA methylation plays a decisive role in controlling the stability, suppressive function, and lineage fidelity of these populations, thereby influencing tumor immune evasion or immune activation [44].

#### Regulatory T cells (Tregs): stability through demethylation of the FOXP3 locus

Tregs are essential for maintaining immune tolerance but become a major barrier to anti-tumor immunity due to their potent suppressive activity within tumors. The master regulator of Treg identity, FOXP3, is controlled by a region known as the Treg-Specific Demethylated Region (TSDR). Stable, lineage-committed Tregs are characterized by complete demethylation of the FOXP3 TSDR, which maintains high and persistent FOXP3 expression even in the face of inflammatory cytokines [54].

In contrast, conventional CD4⁺ T cells that transiently express FOXP3 under inflammatory conditions retain a methylated TSDR and thus lack stable suppressive function. The demethylated TSDR therefore serves as an epigenetic hallmark of committed, suppressive Tregs [99].

Both DNMT1 and TET enzymes participate in preserving or modifying FOXP3 methylation. DNMT1 maintains methylation in non-Treg cells to prevent inappropriate FOXP3 expression, while TET proteins sustain hypomethylation in bona fide Tregs. Manipulation of DNMT or TET activity can therefore alter Treg stability, raising the possibility of therapeutically destabilizing tumor-infiltrating Tregs to enhance anti-tumor immunity [8].

#### CD4⁺ Th1 cells: methylation control of IFNG and TBX21

Th1 cells play a critical protective role in anti-tumor immunity by producing IFN-γ and supporting CD8⁺ cytotoxic responses. Similar to CD8⁺ T cells, the *IFNG* locus undergoes demethylation upon Th1 differentiation, which enables rapid and high-level IFN-γ expression. The promoter of *TBX21* (encoding the Th1 master regulator T-bet) must also be in a hypomethylated and accessible state to drive Th1 commitment [61, 85].

In tumors, however, Th1 differentiation is often impaired. Repressive methylation may become re-established at *IFNG* or *TBX21*, reducing IFN-γ production and weakening the anti-tumor response. Consequently, the Th1/Treg balance within the TME, shaped in large part by DNA methylation, is a key determinant of whether tumors are eradicated or permitted to progress [51].

#### DNA Methylation as an integrator of TME-imposed signals

The TME imposes chronic antigen stimulation, metabolic stress, hypoxia, and suppressive cytokines (TGF-β, IL-10), all of which converge on the epigenetic machinery. Hypoxia, for example, can limit TET activity by reducing α-ketoglutarate availability, favoring hypermethylation and reinforcing exhaustion or Treg stability. Conversely, pro-inflammatory cytokines can increase TET-mediated demethylation at effector loci, although this is often overridden by tumor-induced signals [78].

Thus, DNA methylation functions as a central integrator of competing pro-effector and pro-suppressive inputs, with the net methylation outcome determining T-cell fate and anti-tumor capacity.

### Integrative perspective: DNA methylation as a master regulator of T-cell fate across immune contexts

Across acute infection, chronic infection, and tumorigenesis, DNA methylation governs fundamental T cell fate decisions. During acute infection, rapid and reversible demethylation enables activation of effector programs while preserving the capacity to form memory T cells. In chronic infection, the role of DNMT3A expands to impose a durable, largely irreversible exhaustion program that represses effector genes and enforces sustained expression of inhibitory receptors. In cancer, these same exhaustion pathways are co-opted, while selective methylation remodeling simultaneously stabilizes Treg identity and impairs Th1 differentiation.

The dynamic interplay between DNMTs and TET enzymes thus creates a spectrum of T-cell methylation states, ranging from naïve quiescence to effector activation, memory poising, exhaustion, and regulatory suppression. Understanding these states provides insight into why T cells effectively control acute infections yet fail in chronic diseases and cancer, and it unveils opportunities for therapeutic intervention.

### Therapeutic implications of DNA methylation in T cell-based therapies

A major obstacle in adoptive T cell therapies, including CAR-T and TCR-engineered T cells, is T cell exhaustion. This dysfunctional state is characterized by diminished effector function and persistent expression of inhibitory receptors such as *PDCD1,* TIM-3, and LAG-3 [14, 68]. Epigenetically, exhaustion is marked by hypermethylation of effector gene loci and hypomethylation of inhibitory receptor genes like *PDCD1*, resulting in stable repression or activation, respectively. Preclinical studies show that disrupting DNA methyltransferase (DNMT) activity, or treating with DNMT inhibitors such as decitabine or azacitidine, can reverse these epigenetic marks, restore cytokine production, and reinvigorate exhausted T cells [11, 29, 32, 42, 87]. Notably, preconditioning T cells with DNMT inhibitors prior to CAR transduction enhances their proliferative capacity and persistence, and combining such agents with immune checkpoint blockade may yield synergistic therapeutic effects (see Table 3).

**Fig. 1:**
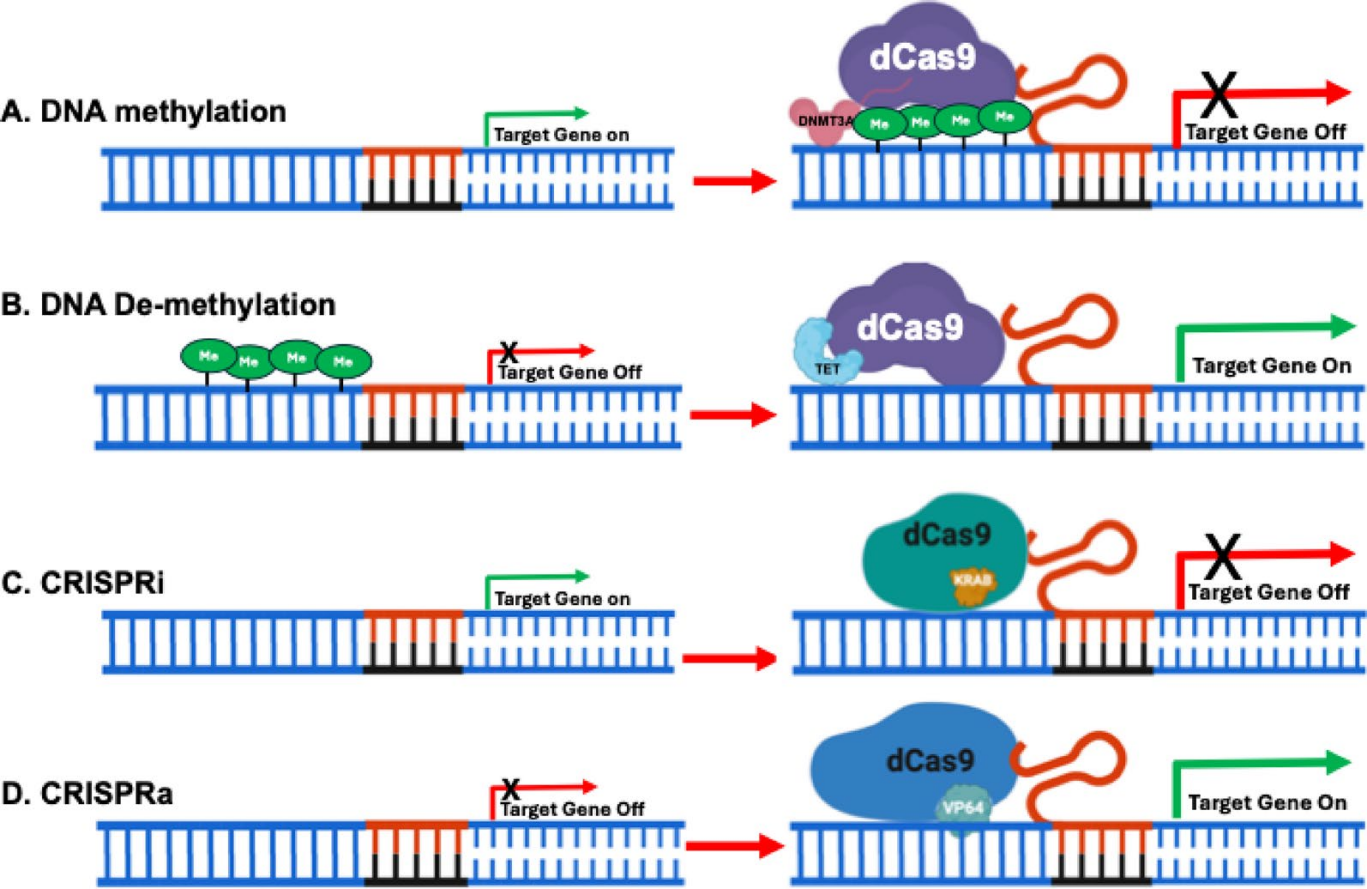
Enhancing T Cell-Based Therapies Using CRISPR-Cas to Target Epigenetic Regulators. In the CRISPR-dCas9 system, an enzymatically inactive dCas9 is fused with functional domains such as DNA methyltransferase 3A (DNMT3A), Ten-Eleven Translocation dioxygenase 1 (TET1), Krüppel-associated box (KRAB), or transcriptional activation domains like VP64. These fusions enable the activation or repression of specific genes. A single-guide RNA (sgRNA) is used to direct the dCas9 complex to the target DNA sequence, ensuring precise gene modulation. **A** dCas9 is fused with DNA methyltransferase 3A (DNMT3A) to enable precise de novo methylation of specific gene promoters; **B** dCas9 is fused with Ten-Eleven Translocation dioxygenase 1 (TET1) to enable the removal of DNA methylation from targeted gene promoters; **C** dCas9 is fused with the Krüppel-associated box (KRAB) domain to repress specific target genes; and **D** dCas9 is fused with the transcriptional activation domain VP64 to activate specific target genes

Beyond reversing exhaustion, modulating DNA methylation can also promote the formation and maintenance of memory T cells, which are essential for durable antitumor immunity. Memory T cells typically exhibit hypomethylation at genes encoding key cytokines and transcription factors such as *IL-2*, *TCF7*, and *FOXP3*, supporting their longevity and functional potential [108, 131]. Emerging epigenetic editing technologies, like CRISPR-dCas9 fused with TET enzymes, allow for locus-specific demethylation of genes such as *TCF7*, thereby enhancing its expression and driving memory T cell differentiation [29, 42, 62]. This approach has the potential to improve the persistence and efficacy of engineered T cells following infusion into patients (see Fig. 1, Table 1 and Table 3).

**Table 1:** Key enzymes involved in DNA methylation. mRNA Methylation, and histone methylation

Methylation types	Enzymes (writers)	Erasers (demethylases)	Readers
DNA methylation	DNMT1, DNMT3A. DNMT3B, DNMT3L	TET1, TET2, TET3	MeCP2. MBD1-4, UHRF1, ZBTB33 (Kaiso)
mRNA methylation	METTL3, METTL14, WTAP, METTL16	FTO, ALKBH5	YTHDF1-3, YTHDC1-2, IGF2BP1-3
*Histone methylation*			
H3K4	SET1/MLL complexes (e.g., MLL1-5, SETD1A/B)	LSD 1, KDM5 family	CHD1, TAF3, ING family
H3K9	SUV39H1/2, SETDB1, G9a/GLP	KDM3 (JMJD1), KDM4 (JMJD2)	HP1 proteins (CBX family)
H3K27	EZH1, EZH2 (PRC2 complex)	KDM6A (UTX), KDM6B (JMJD3)	CBX7.PHF19. EED
H3K36	SETD2, NSD1-3, ASH1L	KDM2A/B	MSH6, LEDGF
H3K79	D0T1L	None identified	L3MBTL2, BPTF
H4K20	SUV4-20H1/2	PHF8, KDM7 family	53BP1, L3MBTL1

Effective trafficking and infiltration of CAR-T and TCR engineered T cells into tumor sites are also crucial for therapeutic success. Tumor-induced hypermethylation of genes encoding chemokine receptors and adhesion molecules, such as *CXCR3* and *CCR5*, impairs T cell migration and limits antitumor activity. DNMT inhibitors can restore the expression of these trafficking-related genes, promoting T cell homing to the tumor microenvironment (TME). Such strategies are particularly valuable in cancers where immune exclusion is a key barrier to effective immunotherapy [31].

The cytotoxic function of T cells depends on effector molecules like perforin, granzyme B, and IFN-γ, whose expression is also regulated by DNA methylation. Tumor cells exploit this by hypermethylating the promoters of *PRF1*, *GZMB*, and *IFNG*, leading to their silencing and reducing T cell-mediated killing [119]. Treatment with DNMT inhibitors has been shown to upregulate these genes, enhancing cytotoxic responses and bolstering the antitumor efficacy of adoptive T cell therapies.

Finally, DNA methylation contributes to immune evasion by tumor cells through the silencing of tumor-associated antigens (TAAs) and MHC class I molecules, which are critical for T cell recognition. Promoter hypermethylation of TAA genes such as *MAGEA1*, *NY-ESO-1*, and *SSX* family members, as well as MHC genes, leads to diminished antigen presentation and reduced immunogenicity in melanoma and lung cancer. DNMT inhibitors can reverse this silencing, reactivating TAA and MHC expression and enhancing tumor visibility [73]. These agents also counteract the immunosuppressive effects of TGF-β in the TME, preserving expression of cytotoxic genes such as *GZMB* and *IFNG* [11]. By integrating DNMT inhibitors with T cell-based therapies, it may be possible to overcome tumor-mediated immunosuppression and improve therapeutic outcomes. Collectively, these strategies highlight the therapeutic potential of targeting DNA methylation to enhance the functionality, persistence, and tumor-targeting capabilities of T cell-based immunotherapies (see Fig. 1, Table 1, Table 2 and Table 3).

**Table 2:** T cell target genes regulated by DNA, mRNA, and histone methylation

Target gene names	Function in T cells	Regulatory mechanism	References
DNA methylation			
BCL6	Germinal center Tfh cell differentiation	Demethylation regulates expression in follicular helper T cells	Nishizawa et al^140^
CD28	Costimulatory signaling molecule	Hypermethylation reduces expression in aging and exhausted T cells	Thomas et al. [108]
CD40L	Costimulatory molecule for T cell help	Promoter demethylation enhances CD40L expression in activated T helper cells	Lian et al.^141^
CTLA4	Immune checkpoint regulator	Hypermethylation suppresses expression, modulating T cell activation	Hoffmann et al. 142
CXCL10	Chemokine involved in T cell migration	DNA methylation repress the tumor production of Th1-type chemokines CXCL9 and CXCL10	Peng et al. 143
CYLD	Negative regulator of NF-κB signaling	Hypermethylation reduces expression, promoting T cell activation	Schott et al. ( 144
EOMES	Effector and memory T cell differentiation	Promoter methylation regulates expression levels in T cells	Zhang et al. 145
FASLG (FasL)	Induces apoptosis in target cells	Promoter demethylation increases expression during T cell-mediated cytotoxic responses	Castellano et al. 146
FOXP3	Regulatory T cell (Treg) differentiation and function	Demethylation of TSDR (Treg-specific demethylated region) is essential for stable Treg function	Bai et al. [8]
GZMB	Cytotoxic granzyme expression	Promoter methylation affects cytotoxic T cell activity	Scharer et al. [93]
HIF1A	Regulator of T cell metabolism	DNA methylation enhances stability and translation during hypoxic stress	Kim et al. 147
IFNG (IFN-γ)	Cytokine production	Promoter demethylation enhances IFN-γ expression, critical for effector T cell responses	Yano et al. 148
IL2	T cell proliferation and survival	Demethylation of regulatory regions increases expression in activated T cells	Teghanemt et al. 149
IL7R	T cell survival and homeostasis	Promoter methylation regulates IL-7 receptor expression, critical for naïve and memory T cells	Shin et al. 150
LAG3	Immune checkpoint receptor	Hypermethylation silences expression, regulating T cell activation	Klümper et al. 151
PDCD1 (PD-1)	Inhibitory receptor involved in T cell exhaustion	Hypomethylation in exhausted T cells increases expression, impacting immune checkpoint pathways	Wang et al. [116]
RORC (RORγt)	Th17 cell differentiation	Promoter demethylation enhances expression, crucial for Th17 lineage specification	Qiu et al. 152
TBX21 (T-bet)	Master regulator of Th1 differentiation	Demethylation promotes T-bet expression, driving Th1 lineage commitment	Qiu et al. 152
TET2	Epigenetic regulator of T cell function	Mutations or reduced expression via methylation affects DNA demethylation processes	Ichiyama et al. 153
TOX	Regulator of T cell exhaustion	Hypomethylation upregulates TOX in exhausted T cells, sustaining their persistence	Rodriguez et al. 154
mRNA methylation (m6A)			
4-1BB	Regulates mRNA stability and translation	m6A-mediated regulation affects stability and translation during T cell activation	Tomasik et al. [110]
CD28	Regulates mRNA stability and translation	m6A-mediated regulation affects stability and translation during T cell activation	Tomasik et al. [110]
CTLA-4	Regulates mRNA stability and translation	silencing of METTL3 or METTL14 reduces CTLA-4 levels	Buchbinder and Desai [12]
*EOMES*	Regulates mRNA stability and translation	FTO and ALKBH5 demethylate mRNA of EOMES, enhancing memory T-cell formation	Jiang et al. [46]
IFN-γ	m6A amplifies Effector Programs & Regulates Memory	m6A deposition enhances translation efficiency, boosting immediate cytotoxicity	Wang et al. [115]
IL-2	Regulates mRNA stability and translation	m6A-mediated regulation affects stability and translation during T cell activation	Yang et al. [126]
PD-1	Regulates mRNA stability and translation	FTO demethylates mRNA of PD-1, and suppress PD-1 expression	Su et al. [104]
*TCF7*	Regulates mRNA stability and translation	FTO and ALKBH5 demethylate mRNA of TCF7, enhancing memory T-cell formation	Jiang et al. [46]
Tim-3	Regulates mRNA stability and translation	m6A-mediated regulation affects stability and translation during T cell activation	Chao et al. [13]
Histone methylation			
FASL	Effector CD8⁺ T cells	activated by marks H3K4me3	Henning et al. [36]
FOXP3	Regulatory T cells (Tregs) in the TME	activated by marks H3K4me3	Floess et al. [26]
*GATA3*	Effector T-helper cell (Th1, Th2, Th17)	repressed by H3K9me3	Wei et al. 155
*GZMB*	CD8⁺ TILs, Effector CD8⁺ T cells	repressed by H3K27me3 and H3K9me3	Lu et al. [72]
*GZMK*	Effector CD8⁺ T cells	activated by marks H3K4me3	Henning et al. [36]
*HAVCR2* (TIM-3)	exhausted and memory T cells	activated by H3K4me3 and H3K36me3 in exhausted and memory T cells	Sen et al. [96]
*IFNG*	Regulatory T cells (Tregs) in the TME	repressed through H3K27me3 and H3K9me3	Wang et al. [114]
*IL2*	Regulatory T cells (Tregs) in the TME	repressed through H3K27me3 and H3K9me3	Lu et al. [72]
*IL4*	Effector T-helper cell (Th1, Th2, Th17)	repressed by H3K9me3	Wei et al. [117]
*LAG3*	exhausted and memory T cells	activated by H3K4me3 and H3K36me3 in exhausted and memory T cells	Sen et al. [96]
NR4A	Tex​ cells in TILs	activated by marks H3K4me3 and H3K36me	Huang et al. [40]
*PDCD1* (PD-1)	exhausted and memory T cells	activated by H3K4me3 and H3K36me3 in exhausted and memory T cells	Sen et al. [96]
*PRF1*	CD8⁺ TILs	repressed by H3K27me3 and H3K9me3	Vemulawada et al. [113]
*TBX21* (T-bet)	Effector T-helper cell (Th1, Th2, Th17)	activated by marks H3K4me3 and H3K36me	Wei et al. [117]
TOX	Tex cells in TILs	activated by marks H3K4me3 and H3K36me	Huang et al. [40]

**Table 3:** Epigenetic targets with potential to enhance CAR-T cell cancer therapies

TARGET CATEGORY	Target Genes/ Enzymes	Function Mechanism	VALIDATION STATUS	Clinical trial(s) * or pubmed references
DNA METHYLATION	DNMT1, DNMT3A/B	Pre-treatment of T-cells with DNMT inhibitors (e.g., Decitabine) to promote a memory-like phenotype, enhancing CAR-T persistence and reducing exhaustion	Human Phase I/II clinical trial	NCT04337606
	DNMT1	DNMT1 inhibition or depletion reprograms T cells and CAR-T cells into NK-like cells with more robust antitumor effects than parental CAR-T cells. Combined DNMT1 and EZH2 inhibition further potentiated this effect	In vitro (Human & Mouse DNMT1 protein)	PMID: 40117344
	DNMT3A	Deleting the DNMT3A gene in human CAR T cells universally prevented T-cell exhaustion and significantly enhanced anti-tumor activity and persistence	human/ mouse cells in the lab (in vitro)	PMID: 34788079
	DNMT1, DNMT3A/B	To evaluate the efficacy and safety of CD19 PD-1/CD28-CAR-T sequential low-dose decitabine in the treatment of relapse or refractory B cell lymphoma	Human Phase I clinical trial	NCT04850560
	DNMT1, DNMT3A/B	Decitabine-primed tandem CD19/CD20 CAR T cells plus epigenetic agents in aggressive r/r B-NHL with huge tumor burden	Human Phase I/II clinical trial	NCT04553393
	DNMT1, DNMT3A/B	CD19-CAR T cells undergo exhaustion DNA methylation programming in patients with acute lymphoblastic leukemia	Human Clinical Data Analysis	PMID: 34852226
	TET2	TET2 downregulation enhances the antitumor efficacy of CD19 CAR T cells in a preclinical model	Preclinical (Mouse xenograft model), In vitro	PMCID: PMC11866829
	TET2	Disruption of TET2 in CAR T cells enhanced tumor control, leading to greater survival of tumor-bearing mice	Preclinical (Mouse models), In vitro	
	TET2	T cell TET2 disruption cuts the breaks on antitumor CAR T cell therapy	Preclinical (Mouse models), In vitro	PMID: 36959018
	TET2	Disruption of TET2 promotes the therapeutic efficacy of CD19-targeted T cells	Preclinical (Mouse models), In vitro	PMID: 29849141
	TET,TET2,TET3	Activation of TET proteins (TET1, TET2, or TET3) using agents like mitoxantrone may be a novel epigenetic anti-cancer therapy	Preclinical (In vitro Leukemia cell lines)	NCT02465060
RNA METHYLATION	FTO	Targeting m6A regulators like FTO represents a promising therapeutic strategy to complement CAR-T cell therapy, specifically by manipulating immune cell reprogramming and overcoming exhaustion	Review/Mechanistic	PMID: 35296338
	IGF2BP1	Combining IGF2BP1 knockdown with anti-GD2 immunotherapy (a standard treatment) induced a synergistic anti-tumor effect in HR-NB, leading to increased effector CD8 T cells and decreased MDSC (immunosuppressive cells) in the tumor	Mouse Model (in vivo)	PMID: 41328513
	METTL3	Inhibiting METTL3 (Catalytic subunit of the m6A methyltransferase complex): Augments tumor immunogenicity and sustains T-cell function. Enhances responsiveness to immune checkpoint blockade by triggering an interferon response and promoting cell cytotoxicity (IFNγ,Gzm). Reverses T-cell exhaustion	Mice Models (Melanoma/Colorectal Adenocarcinoma) & In Vitro (Human/Mouse T cells)	PMC11578931
	METTL3-METTL14	A compound (WD6305) that degrades the METTL3-METTL14 complex cooperated with anti-PD-1 therapy to suppress tumor growth. This highlights METTL14's pathway as a target for enhancing immunotherapy	Mice Model (In vivo) and Clinical Correlation (various cancer types)	PMID: 40865050
	WTAP	WTAP enhances the expression of PD-L1 in colorectal cancer (CRC) cells under hypoxia. It does this by increasing the m6A modification of PD-L1 mRNA, which subsequently stabilizes the transcript via the m6A"reader" protein, IGF2BP2	Mice Model (In vivo) and In vitro (CRC Cell lines)	PMID: 38508217
	YTHDF2	**Inhibition/Deletion of YTHDF2:** Prevents MHC degradation in tumor cells, making them more visible to cells. Its expression in B-cell malignancies promotes ATP synthesis (oncogenic) and **immune evasion**, sensitizing them to **CAR-T cell therapy** when inhibited	Mice (Subcutaneous Tumor Model), In Vitro (Cell Lines), Review/Immune Cells	PMID:40092843
Histone METHYLATION	DOT1L	DOT1L is crucial for maintaining the naïve state of CD8 + T-cells and preventing premature differentiation. Its inhibition can attenuate GvHD while preserving the antitumor efficacy of T-cells, indicating its potential in adoptive immunotherapy and other immune-mediated disorders	Preclinical (General T-cell function)	PMID: 41169259
	EZH2	Pembrolizumab and Tazemetostat following ASCT or CAR-T for Aggressive B-Cell Non-Hodgkin's Lymphoma (NHL)	Human Phase II clinical trial	NCT06242834
	EZH2	EZH2 inhibition upregulates the tumor antigen GD2 expression in Ewing sarcoma cells, which subsequently increased activation and cytolysis by GD2-CAR T cells in vitro	In vitro (Ewing sarcoma cell lines)	PMID: 31005599
	EZH2	EZH2 inhibitors reprogram lymphoma cells to re-express T-cell engagement genes, reduce regulatory T cells, promote memory CAR CD8 phenotypes, and reduce T-cell exhaustion, leading to increased CAR-T recruitment and improved killing	Mouse Models (syngeneic models reflecting human FL and DLBCL)	PMID: 39642889
	EZH2	EZH2 inhibition enhances anti-CD19 CAR-T efficacy by rewiring cancer cells to be more immunogenic, increasing T-cell activation, expansion, and tumor infiltration. Combined EZH1/EZH2 inhibition further boosted CAR-T efficacy and expansion	In vitro (Human B-cell lymphoma, multiple myeloma, AML, sarcoma, ovarian, prostate cell lines), Mouse Models (preclinical models of liquid and solid cancers)	PMID: 39983725
	EZH2	to evaluate the feasibility and safety of giving tazemetostat followed by standard of care CAR T cell infusion in previously treated diffuse large b-cell lymphoma (DLBCL), follicular lymphoma (FL), and mantle cell lymphoma (MCL)	Human Phase I/II clinical trial	NCT05934838
	EZH2	to learn about the safety and effectiveness of the combination of tazemetostat pills in combination with mosunetuzumab injections for people with follicular lymphoma who haven't received treatment before	Human Phase I/II clinical trial	NCT05994235
	G9a/GLP	Short-term inhibition of G9a/GLP during ex vivo T cell expansion increased the antitumor activity of TCR-engineered T cells (a form of adoptive T-cell therapy, similar in concept to CAR-T) in in vitro models and an orthotopic mouse model of hepatocellular carcinoma	Mouse Model & In vitro	PMID: 36732506
	G9a/GLP	Chemical inhibition of G9a/GLP during the differentiation of human induced pluripotent stem cells (iPSCs) facilitates the generation of mature iPSC-derived T cells. CAR-T cells generated this way showed enhanced effector functions in vitro and durable, persistent antitumor activity in a xenograft tumor-rechallenge model (mouse model)	Mouse Model & In vitro	PMID: 39504968
	G9a/GLP	G9a/GLP inhibition during ex vivo lymphocyte expansion increases in vivo cytotoxicity of engineered T cells against hepatocellular carcinoma	Mouse Model & In vitro	PMID: 36732506
	LSD1	Novel CAR-T cells were engineered to simultaneously express an anti-CD19 CAR and a short hairpin RNA (shRNA) targeting LSD1 mRNA. This combined approach resulted in higher killing efficiency in cell assays	In Vitro Cell Assays (Human CD19-CAR T cells co-cultured with tumor cells)	PMID: 35046962
	LSD1	LSD1 inhibitors used in vitro during T-cell activation/expansion reshape the T-cell epigenome to promote a memory phenotype. This leads to improved resistance to exhaustion and enhanced persistence after adoptive transfer	In Vitro (Human CD19-CAR T cells, Human CD8 + T cells) and Mice Model (OT1 cells in melanoma)	PMID: 39191730
	NSD2	NSD2 was shown to promote the upregulation of the CD38 antigen in Multiple Myeloma (MM) cells, especially in t(4;14) MM. Combining the drug All-trans retinoic acid (ATRA) with anti-CD38 CAR T-cell therapy significantly augmented the CAR-T efficacy. NSD2's role suggests it is an upstream regulator influencing the target recognized by the CAR-T therapy	In vitro (MM cells), Mice model (MM graft model in NOD mice)	PMID: 36918219
	53BP1	TP53 mutations (the gene coding for p53, which 53BP1 interacts with) in cancers like CLL are linked to reduced T-cell transplantation efficacy and diminished anti-CD19 CAR-T cell treatment effectiveness	Mice Model (study showing TP53 mutations in CLL mice reduce anti-CD19 CAR-T efficacy)	PMID: 34830747
	SUV39H1	SUV39H1 inactivation enhances the long-term persistence and stem/memory differentiation of 41BB-based CAR-T cells. Protected **mice** against tumor relapses/rechallenges in lung and disseminated solid tumor models. Single-cell transcriptomic and chromatin analyses of tumor-infiltrating CAR-T cells were performed	Mouse Model & In vitro	PMID: 37934001
	SUV39H1	Genetic disruption of SUV39H1 enhances early expansion, long-term persistence, and anti-tumor effect of CAR-T cells. This effect was observed in in vitro and in vivo models (leukemia and prostate cancer)	Mouse Model & In vitro	PMID: 375456717
	SUV39H1	SUV39H1 expression was elevated in human colon carcinoma and tumor-infiltrating CTLs in a mouse colon carcinoma model. Inhibition of SUV39H1 with a small molecule (F5446) increased CTL effector gene expression and suppressed colon carcinoma growth in vivo. (While not CAR-T, it supports SUV39H1 as an immunotherapy target)	Mouse Model & In vitro	PMID: 30610059

^*^ https://clinicaltrials.gov

## Roles of mRNA methylation in infection and tumorigenesis

Messenger RNA (mRNA) modifications constitute an essential regulatory layer that fine-tunes gene expression beyond the DNA sequence. An mRNA modification is a chemical alteration, most often a methylation or other covalent change, added to a nucleotide base or the ribose sugar after transcription. In contrast to canonical features such as the 5′ cap (7-methylguanosine) and poly(A) tail, which are nearly universal in mature eukaryotic mRNAs, internal mRNA modifications are selectively installed within coding regions and untranslated regions (UTRs) [98]. These internal marks form the basis of “epitranscriptomics,” an emerging field focused on how chemical signatures expand the regulatory capacity of the transcriptome. By layering information on top of the RNA sequence, these modifications function as an “RNA code” that influences stability, translation, splicing, and decay, ultimately shaping cellular identity and function.

Among the more than 170 identified RNA modifications, N6-methyladenosine (m6A) is the most abundant and best-characterized internal mark on mRNA, particularly in immune regulation. m6A dynamics are controlled by three groups of proteins, writers, erasers, and readers, which together determine where modifications are placed, removed, and interpreted [39, 56].

The canonical m6A writer complex consists of the catalytic enzyme METTL3, its structural partner METTL14, and the regulatory subunit WTAP, which cooperatively methylate adenosine residues within the DRACH consensus motif. Other mRNA modifications are installed by distinct writer enzymes, such as NSUN2 for 5-methylcytosine (m5C) and TRMT6/TRMT61A for N1-methyladenosine (m1A) [69, 109, 112]. These enzymes act co-transcriptionally or shortly after transcription to shape the emerging transcriptome.

The m6A mark is removed primarily by the α-ketoglutarate-dependent dioxygenases FTO and ALKBH5, which oxidize and demethylate modified adenosines. Additional demethylases, such as **T**ET1, have been implicated in removing m5C from RNA [125]. Through these erasers, cells rapidly reconfigure their transcriptomes in response to environmental signals, enabling fine temporal control of T cell activation and differentiation [45, 136].

The best-studied m6A readers belong to the YTH domain family: YTHDF1 enhances translation, YTHDF2 promotes mRNA decay, and YTHDF3 cooperates with the other two to regulate RNA fate. Nuclear readers, such as YTHDC1, regulate splicing and nuclear export. Other proteins, including ALYREF and YBX1 for m5C and additional YTH-domain proteins for m1A, bind their respective marks to modulate RNA processing. Through these interactions, mRNA modifications become interpretable signals that shape gene networks essential for immunity [82].

Together, the writers, erasers, and readers of m6A establish a dynamic and responsive epitranscriptomic system. In T cells, this system orchestrates rapid transcriptional and translational remodeling during activation, clonal expansion, and fate specification, key processes in immune responses to both infection and tumorigenesis.

T cells undergo extensive transcriptional reprogramming upon antigen recognition, differentiating into short-lived effector T cells (Teff), long-lived memory T cells (Tmem), or exhausted T cells (Tex) under chronic stimulation. mRNA modifications, especially m6A, regulate these developmental transitions by modulating the stability and translation of transcripts encoding transcription factors, cytokines, and signaling molecules that determine T cell fate.

### mRNA methylation in acute infection: coordinating effector responses and memory formation

During an acute, self-limited infection, naïve CD8⁺ T cells rapidly proliferate and differentiate into cytotoxic effector cells that eradicate pathogens. After antigen clearance, most effector cells die, while a subset transitions into long-lived memory T cells.

m6A modifications act as rapid amplifiers of effector programs. Upon activation, T cells increase writer and reader activity. m6A deposition on transcripts encoding key cytokines, such as IFN-γ and IL-2, or signaling molecules enhances translation efficiency, enabling swift deployment of effector functions. By accelerating translation and modulating decay, m6A ensures the timely emergence of high-functioning cytotoxic lymphocytes [115, 137].

The transition to memory requires a distinct transcriptional program. The m6A machinery facilitates this shift by regulating transcripts encoding memory-associated transcription factors such as TCF7. Altered expression or activity of the eraser ALKBH5 can influence TCF7 mRNA stability, adjusting the balance between terminal effector differentiation and memory formation [127].

Thus, during acute infection, mRNA modifications fine-tune both the magnitude of effector responses and the quality of memory formation, balancing immediate defense with long-term protection.

### mRNA methylation in chronic infection: stabilizing the exhausted T cell state

In chronic viral infections (e.g., HIV, HCV) or experimental chronic LCMV, persistent antigen exposure drives T cells into exhaustion, a dysfunctional state characterized by reduced cytokine production, impaired cytotoxicity, sustained inhibitory receptor expression (PD-1, TIM-3), and extensive transcriptional and epigenetic remodeling [9].

m6A-dependent pathways enforce this fate. Dysregulation of writers or readers destabilizes transcripts required for effector function while stabilizing or preferentially translating transcripts promoting exhaustion. Some m6A readers accelerate decay of activation- or proliferation-related mRNAs, whereas others support expression of inhibitory receptors or exhaustion-associated transcription factors.

This epitranscriptomic reprogramming reinforces the exhausted state despite ongoing inflammatory stimuli, thereby distinguishing chronic from acute T cell responses.

### mRNA methylation in tumorigenesis and anti-tumor immunity

mRNA modifications also influence the tumor microenvironment (TME), affecting both cancer cells and infiltrating T cells. Cancer cells frequently exploit mRNA modifications to enhance proliferation, survival, and metastasis. Aberrant activities of m6A writers, erasers, or readers dysregulate expression of oncogenes and tumor suppressors. Within the TME, tumors create immunosuppressive conditions that drive infiltrating T cells them toward exhaustion-like states reminiscent of those in chronic infection [25].

In tumor-infiltrating lymphocytes (TILs), m6A functions as a checkpoint regulator influencing cytotoxicity, cytokine production, and inhibitory receptor expression. Upregulation of certain m6A regulators promotes decay of effector-associated transcripts while stabilizing exhaustion-related transcripts, leading hyporesponsive T cells that fail to eliminate cancer cells [66].

This epitranscriptomic remodeling sustains an immunosuppressive microenvironment and undermines endogenous anti-tumor immunity. The mechanism closely parallels those in chronic infections, reflecting shared principles of persistent antigen exposure.

In summary, mRNA modifications operate as a master regulatory layer that rapidly and reversibly shapes cellular gene expression programs. By controlling the stability, translation, and fate of key transcripts, the epitranscriptome plays essential, and often opposing, roles in T cell differentiation during acute infection, chronic disease, and cancer. These insights highlight new opportunities to therapeutically manipulate RNA modifications to enhance immunity while limiting dysfunction.

### Therapeutic implication of mRNA methylation in T cell-based therapies

Targeting mRNA methylation, particularly N6-methyladenosine (m6A) modifications, presents a promising strategy to enhance CAR-T cell function. Regulators such as METTL3, METTL14, FTO, and ALKBH5 play critical roles in controlling the translation and stability of mRNAs encoding cytokines, co-stimulatory molecules, immune checkpoint receptors, and memory-related transcription factors [16, 35, 74, 104]. Details can be found Tables 1, 2 and 3. Modulating these pathways can increase the translation of IL-2, IFN-γ, CD28, and 4-1BB, thereby boosting CAR-T cell activation, proliferation, and persistence in the tumor microenvironment. Enhancing IL-2 expression through m6A regulation, for example, can promotes CAR-T cell expansion and strengthens anti-tumor responses [126].

A key barrier to durable CAR-T efficacy is T-cell exhaustion, characterized by high expression of inhibitory receptors such as PD-1 and Tim-3. m6A methylation influences the expression of these exhaustion markers [13]. Inhibition of METTL3 or activation of demethylases like FTO can stabilize transcripts essential for effector functions and reduce exhaustion-related gene expression, enhancing CAR-T cell survival and cytotoxicity. Conversely, in some contexts, FTO inhibition may also suppress PD-1 expression and reinvigorate exhausted T cells, suggesting that precise modulation of m6A regulators must be context-dependent [104]. Although METTL3 and FTO have opposing enzymatic functions, inhibiting either can similarly rejuvenate T cells. This apparent paradox arises from the transcript-specific nature of m⁶A regulation: many exhaustion-promoting mRNAs rely on precise m⁶A-dependent stability. METTL3 inhibition reduces m⁶A deposition on these transcripts, while FTO inhibition blocks their demethylation, accelerating their decay. In both cases, the result is decreased expression of key drivers of exhaustion, thereby enhancing T-cell function [118].

Beyond T-cell intrinsic regulation, mRNA methylation also shapes immune checkpoint pathways and tumor antigen expression. In CAR-T cells, silencing METTL3 or METTL14 reduces PD-1 and CTLA-4 levels, promoting resistance to tumor-induced suppression [12]. In tumor cells, targeting mRNA methylation regulators offers a strategy to restore or enhance the expression of tumor-specific antigens, which are often downregulated to evade immune surveillance. For instance, the YTHDF family of m6A reader proteins, YTHDF1, YTHDF2, and YTHDF3, modulates mRNA translation and decay, directly influencing antigen expression critical for immune recognition [46, 65, 74]. YTHDF1 enhances translation of m6A-modified transcripts, while YTHDF3 partners with YTHDF1 to promote translation and with YTHDF2 to accelerate decay. Dysregulation of writers like METTL3 or erasers such as FTO can impair antigen presentation, further contributing to immune escape. By adjusting these pathways, expression of silenced tumor antigens such as NY-ESO-1 or MAGE-A3 can be reactivated, allowing for improved CAR-T cell recognition and cytotoxicity. This represents a novel intersection of epigenetic and adoptive cell therapy strategies to overcome immune resistance [88].

m6A regulation is also essential for memory T-cell formation. FTO and ALKBH5 demethylate transcripts of genes like *TCF7* and *Eomesodermin (EOMES),* which are required for generating and maintaining memory-like CAR-T cells [46]. Modulating these pathways enhances T-cell persistence and equips engineered cells with stem-like qualities capable of mounting long-term responses and resisting tumor-induced dysfunction. Altogether, targeting mRNA methylation offers a multifaceted approach to optimize CAR-T therapy—enhancing activation, reducing exhaustion, restoring tumor antigen expression, and sustaining memory cell function for more effective and lasting cancer treatment.

## Histone methylations and their roles in infection and tumorigenesis

In the cell nucleus, DNA is packaged into repeating subunits called nucleosomes. Each nucleosome consists of approximately 147 base pairs of DNA wrapped around a histone octamer, forming the core nucleosome. These positively charged histones stabilize the DNA structure through electrostatic interactions with the negatively charged DNA. The organization of nucleosomes forms chromatin, whose structural dynamics are governed by histone modifications. These chemical modifications regulate the interaction between histones and DNA, thereby altering chromatin accessibility and gene expression [43, 52]. Chromatin exists in two major states: heterochromatin, which is highly condensed and typically transcriptionally silent (e.g., inactive X chromosomes, centromeres, retroelement-rich regions), and euchromatin, which is more open and associated with actively expressed housekeeping and cell-type-specific genes. Among histone modifications, acetylation is one of the most extensively studied and is known to reduce histone-DNA interactions by neutralizing histone charge, thus loosening chromatin structure and promoting transcription factor accessibility. Other modifications, including methylation and phosphorylation, often function in concert with acetylation to modulate chromatin architecture and regulate gene expression [89, 103]. In this review, we focus on histone methylation as a pivotal regulator of gene expression, with a particular emphasis on its role in infection and T cell differentiation and exhaustion.

Histone methylation is a key epigenetic modification involving the addition of one, two, or three methyl groups to lysine or arginine residues on histones. This process alters chromatin structure and thereby modulates gene expression, depending on the specific residue modified and the degree of methylation. Some methylation marks correlate with gene activation, while others are associated with transcriptional repression. For example, trimethylation of histone H3 on lysine 4 (H3K4me3) is a hallmark of active transcription, particularly at gene promoters. This modification is catalyzed by members of the Trithorax group, including Set1 in yeast, the MLL family in mammals, and ASH1L [22]. These enzymes not only deposit H3K4me3 but also collaborate with chromatin remodeling complexes and transcriptional activators to maintain an open chromatin conformation that facilitates transcription. H3K4me3 enhances the recruitment of transcriptional machinery and is essential for initiating and sustaining gene expression at targeted loci.

Another important modification is histone H3K36 methylation (H3K36me), which plays a vital role in T cell biology and shows strong therapeutic potential in T cell-based cancer therapies. This methylation is established by "writer" enzymes such as SETD2, NSD1-3, and ASH1L; reversed by "erasers" like KDM2A/B; and recognized by "readers" including MSH6 and LEDGF. Among these, H3K36me3 is associated with active transcription and supports lineage-specific gene expression essential for T cell development, differentiation, and activation [15, 18]. SETD2-mediated H3K36me3 stabilizes chromatin and suppresses aberrant transcription, while LEDGF recruits transcriptional complexes to key gene loci in T cells [15, 122]. Therapeutically, enhancing SETD2 activity may boost T cell effector functions and anti-tumor responses. Likewise, targeting KDM2A/B to preserve H3K36me3 could enhance memory T cell formation and persistence, improving the efficacy of adoptive cell therapies such as CAR-T cells [120].

Histone H3K79 methylation (H3K79me), uniquely catalyzed by the enzyme DOT1L, also plays a crucial role in T cell function with notable implications for immunotherapy. Unlike other histone marks, H3K79me has no known demethylase, suggesting inherent stability. It is recognized by reader proteins such as L3MBTL2 and BPTF, and is generally associated with active transcription. DOT1L-mediated H3K79me2 is particularly important for regulating genes involved in T cell lineage specification, proliferation, and cell cycle progression, all of which are critical during T cell activation [53]. Therapeutically, DOT1L inhibition may mitigate T cell exhaustion by reprogramming transcriptional networks, thereby enhancing CAR-T cell persistence. Conversely, strategies to increase H3K79me could reinforce effector T cell functions and counteract immunosuppression within the tumor microenvironment [121].

H4K20 methylation (H4K20me), controlled by SUV4-20H1/2 and removed by demethylases such as PHF8 and the KDM7 family, plays a key role in chromatin compaction, transcriptional repression, and genomic stability. Reader proteins like 53BP1 link this mark to the DNA damage response, a process critical for maintaining genome integrity during T cell proliferation. Specifically, 53BP1-mediated recognition of H4K20me facilitates DNA repair under replicative stress [6]. Enhancing the activity of SUV4-20H enzymes could improve genomic stability in T cells, minimizing functional decline during adoptive therapy. Conversely, inhibiting PHF8 or KDM7 may help maintain H4K20me levels, preserving memory T cell function and promoting durable anti-tumor immunity [24].

In contrast, methylation of H3K9 and H3K27 is typically associated with gene repression and the formation of heterochromatin. H3K9 trimethylation (H3K9me3), catalyzed by SUV39H1/2, is a well-known repressive mark. These enzymes, members of the SET domain family, promote the formation of silent chromatin domains by recruiting heterochromatin protein 1 (HP1), which binds to H3K9me3 and reinforces chromatin compaction [30]. Additional enzymes like G9a and GLP contribute to H3K9 methylation in facultative heterochromatin, enhancing transcriptional repression in specific genomic regions [34].

Similarly, H3K27 trimethylation (H3K27me3), catalyzed by EZH2 of the Polycomb Repressive Complex 2 (PRC2), is a key repressive histone mark. PRC2 components, including EZH2, SUZ12, EED, and RBBP4, work together to deposit and maintain this modification [58]. H3K27me3 silences developmental genes and is essential for processes such as X-chromosome inactivation and differentiation. This mark also recruits additional Polycomb group proteins, including CBX proteins, which stabilize the repressive chromatin state and facilitate the long-term silencing of gene expression during development and cell fate decisions.

Beyond the writers of histone methylation, demethylases also play critical roles in modulating gene expression by removing methyl groups from histone tails. These enzymes fall into two main families: the FAD-dependent amine oxidases and the Jumonji C (JmjC) domain-containing demethylases. FAD-dependent demethylases, such as LSD1 (KDM1A), remove mono- and di-methyl groups from lysine residues including H3K4me1/2 and H3K9me1/2. LSD1 contributes to transcriptional repression through demethylation of H3K9me2, as well as transcriptional activation by demethylating H3K4me1/2. JmjC domain-containing demethylases, including KDM2, KDM4, KDM5, and KDM6, can remove tri-methyl marks such as H3K27me3, H3K36me3, and H3K9me3 [106]. Details on these histone methylation regulators can be found in Tables 1, 2, and 3. By dynamically removing repressive or activating marks, these enzymes maintain chromatin plasticity and play essential roles in regulating gene expression during T-cell development, differentiation, and responses to environmental cues.

### Acute infection: histone methylation drives rapid effector differentiation

During acute infection, naïve T cells experience strong antigenic and inflammatory signals that induce a profound chromatin reorganization. The transition from a naïve to an effector phenotype requires rapid activation of cytotoxic or helper gene programs, silencing of alternative lineage fates, and priming of chromatin for robust transcription.

#### Activation and loss of repressive marks

One of the earliest epigenetic events following TCR activation is the rapid loss of repressive histone marks at key effector loci. Genes such as *IFNG* in CD4⁺ Th1 cells or *GZMB* and *PRF1* in CD8⁺ cytotoxic T lymphocytes (CTLs) undergo swift removal of H3K27me3 and H3K9me2/3, catalyzed by demethylases such as KDM6B (JMJD3) [55]. This demethylation opens chromatin and permits rapid deposition of activating marks, particularly H3K4me3 at promoters and H3K36me3 across gene bodies, thereby enabling high-level transcription of cytokines, cytotoxic molecules, and metabolic genes required for clonal expansion [124].

#### Establishing T-cell lineage identity

Effector T-helper subsets (Th1, Th2, Th17) establish stable lineage programs through graded deposition of activating and repressive methylation. For example, Th1 differentiation requires H3K4me3 enrichment at the *TBX21* (T-bet) gene and its downstream target *IFNG*, while H3K27me3 and H3K9me3 accumulate at Th2-associated loci such as *IL4* and *GATA3* [117]. Conversely, Th2 cells exhibit strong H3K4me3 at *IL4* and H3K27me3 at *TBX21* and *IFNG* [37]. These reciprocal changes ensure mutually exclusive transcriptional access to cytokine genes and stabilize lineage commitment even after the removal of antigenic stimulation.

#### Robust effector function and memory formation

Effector CD8⁺ T cells undergo a similar methylation remodeling process. H3K4me3 accumulates across cytotoxic genes (*GZMB*, *GZMK*, *FASL*), while repressive marks are simultaneously deposited at naïve-associated transcription factors. Importantly, a subset of these marks is only partially remodeled in precursor memory cells. Memory precursors retain “bivalent” chromatin (H3K4me3 + H3K27me3) at some effector loci, allowing them to rapidly reactivate cytotoxic programs upon re-challenge [7, 36]. Thus, histone methylation not only drives effector differentiation but also lays the groundwork for long-term immune protection.

### Chronic infection and T-cell exhaustion: repressive histone methylation locks dysfunction in place

In chronic infections such as HIV, HCV, and LCMV, persistent antigen exposure forces T cells into a distinct differentiation trajectory known as exhaustion. Exhausted T cells progressively lose their capacity to proliferate and produce cytokines but persist in a dysfunctional state marked by sustained expression of inhibitory receptors (PD-1, LAG-3, TIM-3). Unlike acute responses, exhaustion involves stable and often irreversible epigenetic reorganization, particularly in histone methylation landscapes.

#### Silencing of effector gene programs

Chronic TCR stimulation leads to the accumulation of H3K9me2/3 and H3K27me3 at effector loci such as *IFNG*, *TNF*, and *IL2*, as well as transcription factors like *T-bet* that normally promote effector differentiation. Enzymes such as G9a (EHMT2) and SUV39H1 are key contributors to this heterochromatinization [72]. Their activity deposits high levels of H3K9me2/3, generating compact chromatin domains that restrict transcription factor binding and RNA polymerase recruitment.

This repressive chromatin is not easily reversible; even after antigen clearance or checkpoint blockade therapy, effector loci often remain inaccessible. Thus, histone methylation acts as a molecular memory of chronic stimulation that stabilizes the exhausted phenotype.

#### Sustained activation of inhibitory receptors

In contrast to effector genes, inhibitory receptor genes such as *PDCD1* (PD-1), *HAVCR2* (TIM-3), and *LAG3* retain strong activating marks including H3K4me3 and H3K36me3. These active methylation signatures maintain their continuous transcription, reinforcing exhaustion. The presence of persistent H3K4me3 at inhibitory loci represents a key distinction between exhausted and memory T cells, whose inhibitory receptor promoters are largely inactive and lack activating methylation [96].

Together, these epigenetic changes form a rigid chromatin architecture that restricts functional flexibility and supports the persistence of an exhausted but not terminally apoptotic T-cell pool.

### Tumorigenesis: histone methylation shapes dysfunction in tumor-infiltrating T cells

Tumors present a unique environment where both cancer cells and infiltrating immune cells undergo profound epigenetic remodeling. TILs, particularly CD8⁺ T cells, often display an exhaustion-like epigenetic state similar to that seen in chronic viral infection. However, the tumor microenvironment (TME) introduces additional layers of complexity, including immunosuppressive cytokines, metabolic deprivation, and suppressor cell populations.

#### Effector gene suppression in tumor-infiltrating T cells

Within tumors, CD8⁺ TILs frequently accumulate high levels of H3K27me3 and H3K9me3 at effector genes such as *IFNG*, *GZMB*, and *PRF1*. This suppressive signature prevents them from mounting strong cytotoxic responses even when antigen-specific recognition remains intact [72, 113]. Because tumors present persistent antigen and strong inhibitory cues (like PD-L1 expression and TGF-β exposure), these repressive methylation patterns become deeply embedded, mimicking chronic infection-induced exhaustion [63].

#### Active marks at inhibitory and exhaustion-related genes

As in chronic infection, TILs show elevated H3K4me3 and H3K36me3 at genes encoding inhibitory receptors. Tumor-induced transcription factors such as TOX and NR4A further cooperate with epigenetic writers to maintain exhaustion-associated chromatin states. This stable epigenetic program explains why many TILs remain unresponsive to checkpoint blockade: the exhaustion architecture is not easily erased despite transient signaling relief [40, 96, 97].

### Regulatory T cells and immunosuppressive histone methylation

Regulatory T cells (Tregs) within the TME are particularly dependent on epigenetic mechanisms for lineage stability. The FOXP3 locus is heavily enriched in activating marks such as H3K4me3, ensuring high and stable expression of this master regulator [26]. Simultaneously, Tregs silence opposing transcriptional programs through H3K27me3 and H3K9me3 deposition at effector cytokine loci (e.g., *IFNG*, *IL2*). The result is a highly stable suppressive phenotype that is resistant to inflammatory cues [114].

Myeloid-derived suppressor cells (MDSCs), which also populate the TME, contribute indirect epigenetic effects by secreting factors that reinforce repressive histone methylation in T cells. Together, these suppressive populations help maintain the dysfunctional state of TILs and the immunosuppressive milieu favorable to tumor growth [50, 92].

### H3K79 methylation and oncogenic pathways

In certain leukemias, such as MLL-rearranged acute leukemia, MLL fusion proteins aberrantly recruit DOT1L to specific loci, driving abnormally high H3K79me2/3 and overexpression of leukemogenic HOXA cluster genes. DOT1L inhibitors have demonstrated efficacy in these cancers and represent an example of successful therapeutic targeting of histone methylation [76, 102]. While this mechanism directly drives cancer cell proliferation, it also indirectly shapes immune responses by altering tumor antigenicity and the inflammatory milieu.

### Therapeutic implications of histone methylation in T cell-based therapies

Histone methylation is a key epigenetic mechanism that shapes T cell activation, differentiation, and persistence, and its therapeutic manipulation offers promising strategies to improve T cell-based cancer immunotherapies. Activating marks such as H3K4 methylation are enriched at promoters of immune effector genes and support robust cytokine production. Enhancing H3K4 methylation by activating MLL complexes or inhibiting demethylases like LSD1 has been shown to restore expression of cytokines such as IFN-γ, TNF-α, and IL-2, which are critical for antitumor responses. For example, Pallavicini et al. demonstrated that LSD1 inhibition in exhausted T cells restored effector cytokine expression and enhanced cytotoxicity against tumor cells, highlighting a potential epigenetic strategy to reverse T cell dysfunction in the tumor microenvironment [83].

In contrast, repressive marks like H3K27me3, catalyzed by EZH2 of the PRC2, silence genes essential for T cell metabolism and differentiation. Elevated H3K27me3 levels have been observed in tumor-infiltrating T cells and are associated with exhaustion. EZH2 inhibitors can reverse this repression, rejuvenate exhausted T cells, and improve their ability to eliminate tumor cells. Hou and Wu (2024) reported that pharmacological inhibition of EZH2 reactivated silenced metabolic and effector genes, resulting in prolonged T cell persistence and increased antitumor efficacy in preclinical models [86, 101, 139].

**Fig. 2:**
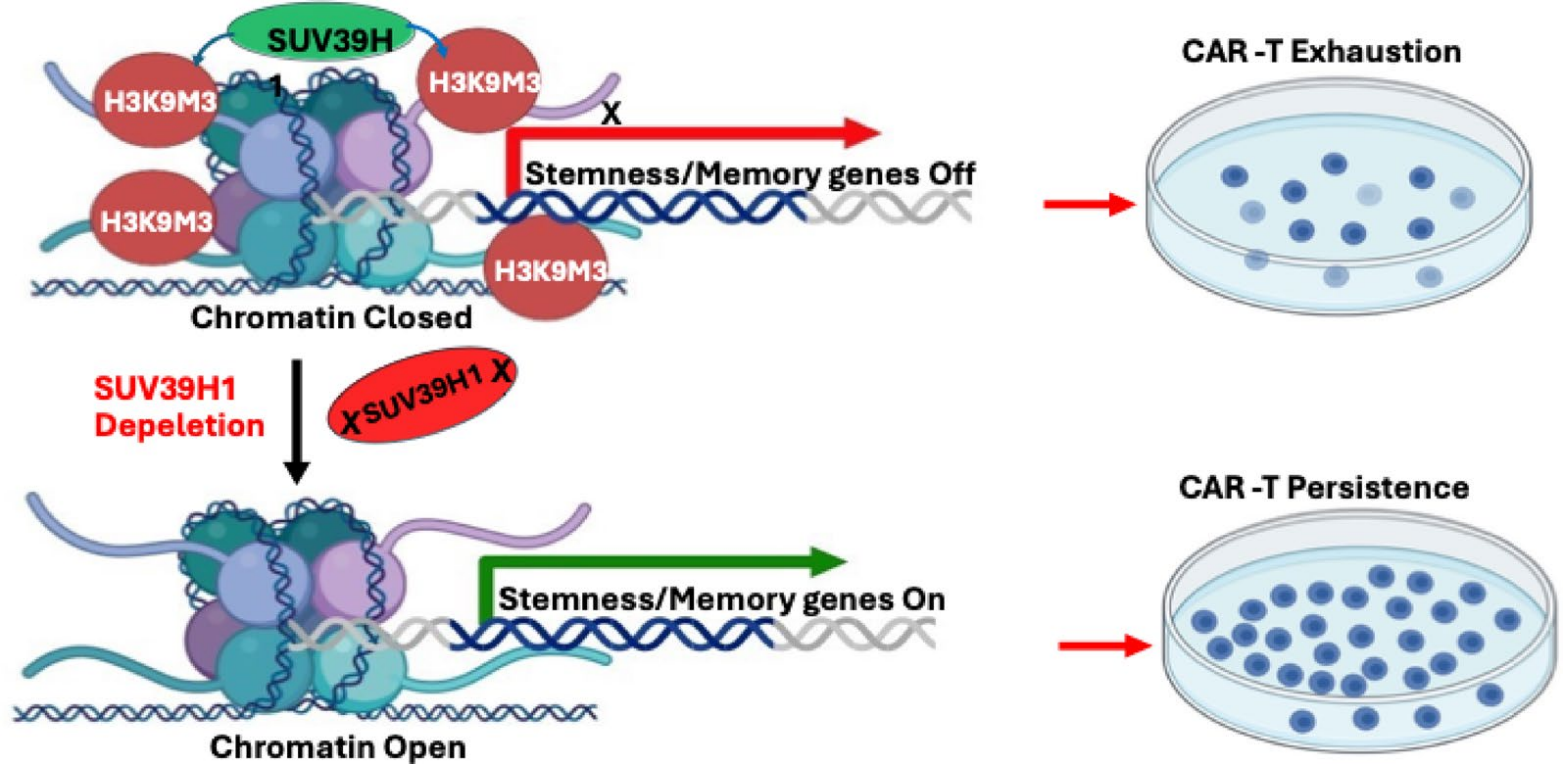
Deletion of SUV39H1 Enhances CAR-T Cell Expansion and Reduces Exhaustion. SUV39H1 catalyzes trimethylation of histone H3 at lysine 9 (H3K9me3), promoting chromatin condensation at stemness- and memory-associated gene loci, thereby contributing to CAR-T cell exhaustion (upper panel). Deletion of SUV39H1 increases chromatin accessibility at these loci, resulting in upregulation of stemness and memory genes, enhanced CAR-T cell expansion, and improved persistence (lower panel)

H3K9 trimethylation, mediated by SUV39H1 and SUV39H2, promotes heterochromatin formation and represses immune effector genes, including those encoding MHC class I molecules and cytotoxic mediators. Inhibition of SUV39H1 enhances CAR-T cell expansion, persistence, and killing capacity, as shown in leukemia and prostate cancer models. It has been shown that SUV39H1 expression in the tumor microenvironment represses cytotoxic T-lymphocyte (CTL) effector genes, such as *GZMB* and *IFNG* by enriching H3K9me3 at their promoters. Inhibiting SUV39H1 with a small molecule inhibitor upregulates these effector genes in CTLs and suppresses tumor growth in a CD8 + CTL-dependent manner [71, 72]. For additional details on the therapeutic potential of targeting SUV39H1 in T cell-based therapy, see Fig. 2 and Table 3.

Additional histone marks, though less extensively studied, also influence T cell responses and may serve as future therapeutic targets. H3K36me3, deposited by SETD2, supports transcriptional fidelity and lineage-specific gene expression during T cell development. Chen et al. showed that SETD2 is crucial for T cell function, acting as an epigenetic regulator that controls T cell activation, differentiation (especially Tregs), survival, and preventing autoimmunity, with its loss linked to T-cell malignancies [15, 17]. Inhibiting KDM2A/B, the demethylases of H3K36me3, may enhance memory T cell formation and persistence, increasing the durability of CAR-T cell therapies.

H3K79 methylation, regulated solely by DOT1L, is associated with transcriptional activation and controls genes involved in T cell proliferation and cell cycle regulation. Inhibiting DOT1L can reprogram exhausted T cells by altering their transcriptional landscape, while enhancing H3K79me2 levels may strengthen T cell responses and reduce immune suppression in tumors [121]. Similarly, H4K20me, deposited by SUV4-20H1/2 and read by proteins like 53BP1, is important for DNA repair and chromatin stability during rapid T cell expansion. Fan et al. [24] suggested that preventing excessive H4K20 demethylation through PHF8 inhibition can preserve memory T cell function and genomic integrity under proliferative stress.

Together, these findings underscore the therapeutic potential of targeting histone methylation to reshape the T cell epigenome, overcome exhaustion, and enhance the effectiveness of adoptive cell therapies. By selectively modulating specific histone marks and their regulatory enzymes, it is possible to fine-tune T cell function and longevity, offering a new dimension of control in next-generation cancer immunotherapy.

## Perspective on epigenetic reprogramming in T cell-based cancer treatment

Epigenetic reprogramming offers a powerful strategy to enhance the efficacy of T cell-based cancer therapies, including CAR-T, TCR-engineered cells, and tumor-infiltrating lymphocytes (TILs). Despite their clinical success, these therapies face challenges such as T cell exhaustion, limited persistence, and resistance within the immunosuppressive tumor microenvironment.

Targeting DNA methylation has shown promise in overcoming immune evasion. DNA methyltransferase (DNMT) inhibitors such as azacitidine (AZA) and decitabine (DAC) can demethylate promoters of tumor-associated antigens (TAAs) and MHC genes to enhance tumor immunogenicity and improve T cell recognition. DAC treatment, for example, restores NY-ESO1 and MAGE-A3 expression in NSCLC models, boosting cytotoxic T cell activity [116]. Moreover, DNMT inhibitors, such as AZA and DCA, have been shown to counteract TGF-β-mediated suppression by preserving expression of key effector genes like *IFNG* and *GZMB*, thereby sustaining T cell function [107].

At the epitranscriptomic level, modulation of mRNA methylation pathways, particularly N6-methyladenosine (m6A), offers dual benefits. Inhibiting m6A demethylases like FTO in tumors may stabilize transcripts encoding pro-inflammatory cytokines, thereby reducing immunosuppressive factors like TGF-β and IL-10 that hinder CAR-T cell activity. Simultaneously, modulating m6A pathways in CAR-T cells can enhance their persistence and cytotoxic function, as seen in preclinical models where m6A readers such as YTHDF1/2 were targeted to improve T cell responses. This dual-targeting strategy that modifies both tumor and CAR-T cell mRNA methylation has the potential to produce a synergistic effect [46]. For example, combining m6A inhibitors with CAR-T cells engineered to resist TGF-β signaling could amplify their therapeutic potency. Additionally, co-administration of m6A-modifying agents might enhance the expression of key chemokines, such as CXCL9 and CXCL10, improving CAR-T cell trafficking to the tumor site. Overall, integrating epitranscriptomic modulation with CAR-T therapy represents a promising avenue for overcoming resistance and achieving more durable responses in cancer treatment.

Histone methylation modifiers also present opportunities for therapeutic synergy. Inhibition of SUV39H1 (H3K9 methyltransferase) or EZH2 (H3K27 methyltransferase) can reverse T cell exhaustion, enhance persistence, and sensitize tumors to immune checkpoint blockade [28, 41]. Integrating these inhibitors with CAR-T or TCR therapies can amplify antitumor responses, particularly when combined with strategies that promote H3K4 methylation or reduce H3K27me3, optimizing T cell function and memory formation.

In the future, epigenetic reprogramming, spanning DNA, histone, and RNA modifications, has the potential to overcome both intrinsic and extrinsic barriers in adoptive T cell therapy. A major challenge will be developing specific and effective strategies tailored to patients with both hematological and solid malignancies.

## Data Availability

Not applicable.
